# Prognostic value of *CCND1* gene status in sporadic breast tumours, as determined by real-time quantitative PCR assays

**DOI:** 10.1038/sj.bjc.6600109

**Published:** 2002-02-12

**Authors:** I Bièche, M Olivi, C Noguès, M Vidaud, R Lidereau

**Affiliations:** Laboratoire de Génétique Moléculaire–UPRES JE 2195, Faculté des Sciences Pharmaceutiques et Biologiques, Université René Descartes–Paris V, 4 Avenue de l'Observatoire, F-75006 Paris, France; Laboratoire d'Oncogénétique–INSERM E0017, France; Département de Stastistiques Médicales, Centre René Huguenin, 35 rue Dailly, F-92211 St-Cloud, France

**Keywords:** breast cancer, *CCND1* expression, quantitative RT–PCR, real-time PCR detection, prognostic value

## Abstract

The *CCND1* gene, a key cell-cycle regulator, is often altered in breast cancer, but the mechanisms underlying *CCND1* dysregulation and the clinical significance of *CCND1* status are unclear. We used real-time quantitative PCR and RT–PCR assays based on fluorescent TaqMan methodology to quantify *CCND1* gene amplification and expression in a large series of breast tumours. *CCND1* overexpression was observed in 44 (32.8%) of 134 breast tumour RNAs, ranging from 3.3 to 43.7 times the level in normal breast tissues, and correlated significantly with positive oestrogen receptor status (*P*=0.0003). *CCND1* overexpression requires oestrogen receptor integrity and is exacerbated by amplification at 11q13 (the site of the *CCND1* gene), owing to an additional gene dosage effect. Our results challenge *CCND1* gene as the main 11q13 amplicon selector. The relapse-free survival time of patients with *CCND1*-amplified tumours was shorter than that of patients without *CCND1* alterations, while that of patients with *CCND1*-unamplified-overexpressed tumours was longer (*P*=0.011). Only the good prognostic significance of *CCND1*-unamplified-overexpression status persisted in Cox multivariate regression analysis. This study confirms that *CCND1* is an ER-responsive or ER-coactivator gene in breast cancer, and points to the *CCND1* gene as a putative molecular marker predictive of hormone responsiveness in breast cancer. Moreover, *CCND1* amplification status dichotomizes the *CCND1*-overexpressing tumors into two groups with opposite outcomes.

*British Journal of Cancer* (2002) **86**, 580–586. DOI: 10.1038/sj/bjc/6600109 www.bjcancer.com

© 2002 Cancer Research UK

## 

Cyclin D1, a protein encoded by the *CCND1* gene, has a well-established role in regulating progression through the G1 phase of the cell cycle. Cyclin D1 acts by complexing with the cyclin-dependent kinases CDK4 and CDK6, promoting phosphorylation and inactivation of retinoblastoma protein. *CCND1* has been identified as an oncogene, and is rearranged, amplified or overexpressed in a variety of tumours (Motokura and Arnold, 1993). Recent results from several groups suggest that cyclin D1 may also be involved in the activities of transcription factors through CDK-independent mechanisms. Cyclin D1 can bind to and regulate the activity of several proteins, including myb-like transcription factor (DMP1) (Inoue and Sherr, 1998), the myogenic transcription factor MyoD (Skapek *et al*, 1996), and also the oestrogen receptor, through the recruitment of p300/CBP-associated protein (P/CAF) and steroid receptor coactivator-1 (SRC-1) (Zwijsen *et al*, 1997, 1998; Neuman *et al*, 1997; McMahon *et al*, 1999).

Cyclin D1 aberrations have been strongly linked to human breast cancer. Ectopic expression of cyclin D1 is sufficient to initiate cell cycle progression in the absence of external growth stimuli (Musgrove *et al*, 1994). Transgenic mice carrying the *CCND1* gene driven by the mouse mammary tumour virus terminal repeat show altered mammary cell proliferation and a high incidence of mammary adenocarcinomas (Wang *et al*, 1994). Clinical studies have found amplification of 11q13 chromosomal region (which contains *CCND1*) in 10–15% of human primary breast cancers (Ali *et al*, 1989; Borg *et al*, 1991; Schuuring *et al*, 1992; Henry *et al*, 1993). However, overexpression (at both the mRNA and protein levels) is seen in about 50% of cases, suggesting that mechanisms other than DNA amplification may dysregulate cyclin D1 expression (McIntosh *et al*, 1995; Gillett *et al*, 1996; Barbareschi *et al*, 1997; Jares *et al*, 1997; Nielsen *et al*, 1997; Kenny *et al*, 1999). It is noteworthy that it has been previously described a high correlation between overexpression of *CCND1* mRNA and increased presence of Cyclin D1 protein (Bartkova *et al*, 1994; Gillett *et al*, 1994).

The regulation of *CCND1* gene expression is poorly understood. Experimental data show that cyclin D1 expression can be regulated by several factors which may be dysregulated in breast cancer, including growth factors (Musgrove *et al*, 1993), p53 through p21WAF1 (Chen *et al*, 1995) and oestrogen (Musgrove *et al*, 1994; Altucci *et al*, 1996). It is noteworthy that most *CCND1*-overexpressing tumours are oestrogen receptor-positive (Hui *et al*, 1996; Barbareschi *et al*, 1997; Jares *et al*, 1997). Finally, cyclin D1 is frequently overexpressed in ductal carcinoma *in situ*, and also in some benign breast diseases (Weinstat-Saslow *et al*, 1995; Alle *et al*, 1998), pointing to a role in the earliest stages of breast tumour development.

The action of cyclin D1 in cell cycle control, its role in murine mammary gland development and oncogenesis, its altered expression in half of all human breast tumours and in the earliest stages of breast oncogenesis, as well as its apparent involvement in the action of oestrogen, have led to numerous studies to ascertain whether cyclin D1 may serve as a biological marker in breast cancer. However, clinical studies have produced unexpected results. Indeed, *CCND1* amplification has been linked to poor outcome (Ali *et al*, 1989; Borg *et al*, 1991; Schuuring *et al*, 1992; Henry *et al*, 1993), whereas overexpression of cyclin D1, as determined by immunohistochemical methods, has been linked to good outcome (Gillett *et al*, 1996). The latter association could be explained by a link between cyclin D1 overexpression and well-differentiated, ER-positive carcinomas, which carry a better prognosis. In this regard, we recently suggested in a small series of breast tumors (*n*=33) that *CCND1* mRNA overexpression is related to oestrogen receptor positively (Spyratos *et al*, 2000).

*CCND1* expression status might also be a useful marker to predict the response to endocrine therapy (Gillett *et al*, 1996; Sutherland *et al*, 1997; Wilcken *et al*, 1997; Barnes and Gillett, 1998).

Finally, *CCND1* appears to be an outstanding candidate therapeutic target, and several studies have shown that antisense to *CCND1* inhibits the growth and reverses the transformed phenotype of human cancer cells (Zhou *et al*, 1995; Arber *et al*, 1997).

These promising clinical perspectives call for a sensitive, accurate and rapid method to screen breast cancer patients for *CCND1* amplification/overexpression. We developed a real-time quantitative RT–PCR assay based on TaqMan methodology to quantify *CCND1* mRNA in homogeneous total RNA solutions obtained from tumour samples (Gibson *et al*, 1996). This method has excellent performance, accuracy and sensitivity, together with a wide dynamic range, a high throughput capacity and good interlaboratory agreement. In addition, it eliminates the need for tedious post-PCR processing.

To determine the prognostic value of *CCND1* amplification and/or overexpression, we used this real-time PCR method to measure *CCND1* gene expression at the mRNA level in a large series of unilateral invasive primary breast tumours (*n*=134) with known *CCND1* gene status (Bièche *et al*, 1998) and available long-term outcome data.

As several studies have pointed to cooperation between the *CCND1* and *RB1* genes, and to their joint involvement in the proliferative capacity of tumour cells, we also sought a possible link between *CCND1* DNA and/or mRNA status and *RB1* mRNA underexpression.

## MATERIALS AND METHODS

### Patients and samples

We analyzed tissue from excised primary breast tumours of 134 women treated at the Centre René Huguenin from 1977 to 1989. The samples were examined histologically for the presence of tumour cells. A tumour sample was considered suitable for this study if the proportion of tumour cells was more than 60%. Immediately following surgery the tumour samples were stored in liquid nitrogen until RNA extraction.

The patients (mean age 58.3 years, range 34–91) met the following criteria: primary unilateral non metastatic breast carcinoma on which complete clinical, histological and biological data were available; and no radiotherapy or chemotherapy before surgery. The main prognostic factors are presented in Table 1

**Table 1:**
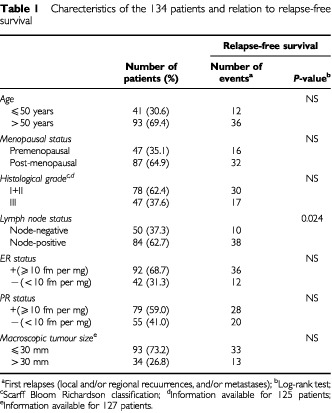
Charecteristics of the 134 patients and relation to relapse-free survival

. The median follow-up was 8.8 years (range 1.0–16.2). Forty-eight patients relapsed (the distribution of first relapse events was as follows: 14 local and/or regional recurrences, 30 metastases and four both).

Specimens of adjacent normal breast tissue from 10 of the breast cancer patients, and normal breast tissue from 10 women undergoing cosmetic breast surgery were used as sources of normal RNA.

### Real-time RT–PCR

#### Theoretical basis

Reactions are characterized by the point during cycling when amplification of the PCR product is first detected, rather than the amount of PCR product accumulated after a fixed number of cycles. The higher the starting quantity of the target molecule, the earlier a significant increase in fluorescence is observed. The parameter C_t_ (threshold cycle) is defined as the fractional cycle number at which the fluorescence generated by cleavage of the probe passes a fixed threshold above baseline. The *CCND1* target message in unknown samples is quantified by measuring C_t_ and by using a standard curve to determine the starting target message quantity.

The precise amount of total RNA added to each reaction mix (based on optical density) and its quality (i.e. lack of extensive degradation) are both difficult to assess. We therefore also quantified transcripts of the gene coding for the TATA box-binding protein (*TBP*) (a component of the DNA-binding protein complex TFIID) as the endogeneous RNA control, and each sample was normalized on the basis of its *TBP* content.

For each experimental sample the amount of the targets and endogeneous reference is determined from the standard curve. Then, the target amount is divided by the endogeneous reference amount to obtain a normalized target value. The relative gene target expression level was also normalized to a normal breast tissue sample (calibrator), or 1×sample. Each of the normalized target values is divided by the calibrator normalized target value to generate the final relative expression levels.

Final results, expressed as N-fold differences in *CCND1* gene expression relative to the *TBP* gene and the calibrator, termed ‘*N_CCND1_*’, was determined as follows:


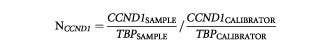


#### Primers, probes and PCR consumables

Primers and probes for the *TBP* and *CCND1* genes were chosen with the assistance of the computer programs Oligo 4.0 (National Biosciences, Plymouth, MN, USA) and Primer Express (Perkin-Elmer Applied Biosystems, Foster City, CA, USA). The primer pairs for *CCND1* were selected to be unique when compared with the sequences of the closely related *CCND2* and *CCND3* genes, and *CCND2PS* and *CCND3PS* pseudogenes. The nucleotide sequences of the oligonucleotide hybridization probes and primers are shown in Table 2

**Table 2:**
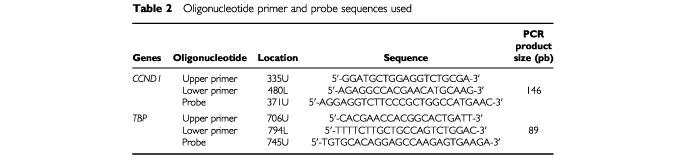
Oligonucleotide primer and probe sequences used

. Primers and Probes are designated by the nucleotide position (relative to *TBP* GenBank Number X54993 and *CCND1* GenBank Number X59798) corresponding to the 5′ position, followed by the letter U for upper (sense strand) or L for lower (antisense strand). To avoid amplification of contaminating genomic DNA, one of the two primers or the probe was placed at the junction between two exons, or in a different exon. For example, the upper primer of *TBP* (706U) was placed in exon 5, the probe (745U) at the junction between exon 5 and exon 6, and the lower primer (794L) were placed in exon 6.

#### RNA extraction

Total RNA was extracted from breast specimens by using the acid-phenol guanidium method (Chomczynski and Sacchi, 1987). The quality of the RNA samples was determined by electrophoresis through denaturing agarose gels and staining with ethidium bromide, and the 18S and 28S RNA bands were visualized under ultraviolet light.

#### Standard curve construction

The relative kinetic method was applied using a standard curve. The latter was constructed with four-fold serial dilutions of total RNA from normal human breast tissues in mouse total RNA (Clontech, Category Number 64042-1). The standard curve used for reverse transcription is composed of five points (1000, 250, 62.5, 15.6 and 3.9 ng of human normal breast total RNA). The series of diluted human total RNAs was aliquoted and stored at −80°C until use.

#### cDNA synthesis

Reverse transcription of RNA was done in a final volume of 20 μl containing 1× RT–PCR buffer (500 mM each dNTP, 3 mM MgCl_2_, 75 mM KCl, 50 mM Tris-HCl pH 8.3), 10 units of RNasin™ Ribonuclease inhibitor (Promega, Madison, WI, USA), 10 mM dithiothreitol, 50 units of Superscript II RNase H^-^ reverse transcriptase (Gibco–BRL, Gaithersburg, MD, USA), 1.5 mM random hexamers (Pharmacia, Uppsala, Sweden) and 1 μg of total RNA (standard curve point samples and patients' samples). The samples were incubated at 20°C for 10 min and 42°C for 30 min, and reverse transcriptase was inactivated by heating at 99°C for 5 min and cooling at 5°C for 5 min.

#### PCR amplification

All PCR reactions were performed using a ABI Prism 7700 Sequence Detection System (Perkin-Elmer Applied Biosystems). For each PCR run a master mix was prepared on ice with 1×TaqMan buffer, 5 mM MgCl_2_, 200 mM dATP, dCTP and dGTP and 400 mM dUTP, 300 nM each primer, 150 nM probe and 1.25 units of AmpliTaq Gold DNA polymerase (Perkin-Elmer Applied Biosystems). Ten microliters of each appropriate diluted RT sample (standard curve points and patients' samples) was added to 40 μl of the PCR master-mix. The thermal cycling conditions comprised an initial denaturation step at 95°C for 10 min and 50 cycles at 95°C for 15 s and 65°C for 1 min.

Experiments were performed with duplicates for each data point. All the patients' samples with a CV of the number of *TBP* or *CCND1* mRNA copies higher than 10% were retested.

### Statistical analysis

Relapse-free survival (RFS) was determined as the interval between diagnosis and detection of the first relapse (local and/or regional recurrences, and/or metastases). Clinical, histological and biological parameters were compared using the chi-square test. Differences between the two populations were judged significant at confidence levels greater than 95% (*P*<0.05). Survival distributions were estimated by the Kaplan–Meier method (Kaplan and Meier, 1958), and the significance of differences between survival rates was ascertained using the log-rank test. Multivariate analysis using Cox's proportional hazards model (Cox, 1972) was used to assess the independent contribution of each variable to RFS.

## RESULTS

### Validation of the standard curve and dynamic range of real-time RT–PCR

The dynamic range of the *CCND1* real-time RT–PCR assay was wide (at least three orders of magnitude) with samples containing as much as 50 ng or as little as 0.2 ng equivalent total cDNA. A strong linear relationship between the C_t_ and the log of starting copy number was always demonstrated (R^2^⩾0.99). The efficiency of the reaction (E), calculated by the formula: E=10^1/|m|^−1, where m is the slope of standard curve line, was ranged from 90 to 100% for the different assays. All breast tissue samples which were analyzed consistently fell within the calibration curve.

### *CCND1* mRNA level in normal breast tissues

To determine the cut-off point for altered *CCND1* gene expression at the RNA level in breast cancer tissue, the N*_CCND1_* value, calculated as described in Materials and methods, was determined for 20 normal breast tissue RNAs. As this value consistently fell between 0.6 and 1.8 (mean 1.03±0.37 standard deviation), values of three (mean+5 s.d.) or more were considered to reflect overexpression of the *CCND1* gene in tumour RNA samples.

### *CCND1* mRNA level in breast tumour tissues

Among the 134 breast tumour RNA samples tested, 44 (32.8%) showed *CCND1* overexpression. Major differences in the amount of *CCND1* mRNA were observed (N_*CCND1*_ from 3.3 to 43.7); 19 tumours (14.2%) had an expression level three to five times higher than that of normal breast tissue, while 15 tumours (11.2%) contained amounts five to 10 times higher, six tumours (4.5%) 10 to 20 times higher, and four tumours (3.0%) more than 20 times higher. Among the 10 patients in whom both the primary breast tumour and matched normal breast tissue were investigated, *CCND1* expression was far higher in three tumours than in the normal tissue (N_*CCND1*_=8.1, 4.8 and 3.6, compared to 0.9, 1.2 and 0.2, respectively).

### Relationship between the *CCND1* RNA levels and *CCND1* amplification levels

All 134 tumours studied for *CCND1* expression at the RNA level had previously been tested for *CCND1* amplification by Southern blot analysis (unpublished data), and 94 had also been tested (when DNA was still available) with a real-time quantitative PCR assay based on TaqMan technology (Bièche *et al*, 1998). As the TaqMan technology was more sensitive than Southern blotting (Bièche *et al*, 1998), we increased the cut-off for gene amplification in the real-time PCR assay from two to 2.5 to have a total correlation between this latter method and Southern blot analysis. With the new cut-off, 15 (11.2%) of the 134 tumours tested here showed *CCND1* amplification. *CCND1* overexpression was found in all but three of the tumours that showed 11q13 amplification ( Table 3

**Table 3:**
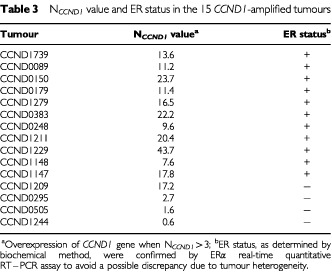
N_*CCND1*_ value and ER status in the 15 *CCND1*-amplified tumours

). The *CCND1* mRNA and DNA status of these three tumours was confirmed by conducting a second RNA and DNA extraction, by additional real-time quantitative PCR and RT–PCR analyses (use of new primer pairs for the *CCND1* and *TBP* genes, and an additional endogeneous RNA control; the *RPLP0* gene (also known as 36B4) encoding human acidic ribosomal phosphoprotein P0) and by Northern and Southern analysis.

Interestingly, the *CCND1*-amplified-overexpressed tumours contained larger amounts of *CCND1* mRNA (12 tumours; mean N_*CCND1*_ 17.9, range 7.6 to 43.7) than did the *CCND1*-unamplified-overexpressed tumours (32 tumours; mean N_*CCND1*_ 5.6; range 3.3 to 8.6).

### Correlation between *CCND1* mRNA and DNA status and clinical, pathological and biological parameters

We sought links between *CCND1* mRNA and DNA status (alteration *versus* normal) and standard clinical, pathological and biological factors in breast cancer ( Table 4

**Table 4:**
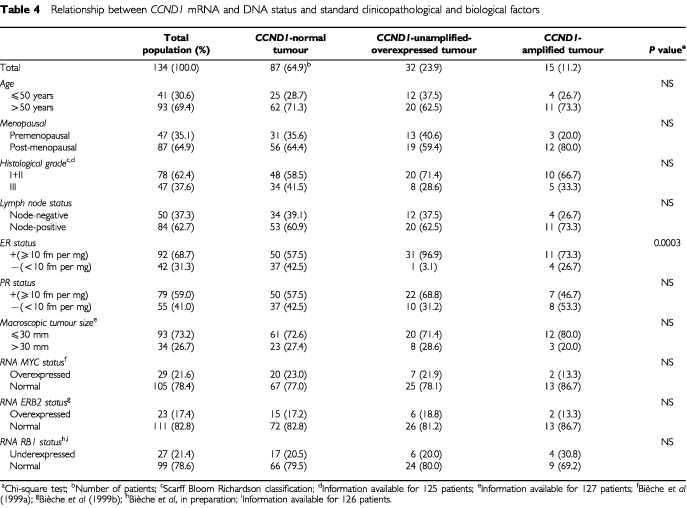
Relationship between *CCND1* mRNA and DNA status and standard clinicopathological and biological factors

). Patients with *CCND1*-altered tumours (*n*=47) were subdivided into those with *CCND1*-amplified tumours (*n*=15) and those with *CCND1*-unamplified-overexpressed tumours (*n*=32).

The only statistically significant link was between *CCND1*-unamplified-overexpressed tumours and oestrogen receptor positivity (*P*=0.0003). Only one (3.1%) of the 32 patients with *CCND1*-unamplified-overexpressed tumours was oestrogen receptor-negative, compared with 41 (40.2%) of the other 102 patients. It should be noted that this isolated tumour had a very low level of *CCND1* overexpression (N_*CCND1*_=3.3).

Neither *CCND1* amplification nor *CCND1* overexpression without amplification was significantly linked to menopausal status or standard prognostic factors such as macroscopic tumour size, histopathological grade and lymph-node or progesterone receptor status.

**Figure 1:**
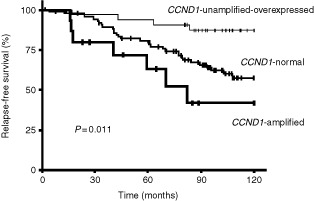
RFS curves for patients with *CCND1*-unamplified-over expressed, *CCND1*-amplified and *CCND1*-normal tumours.

Univariate analysis (log-rank test) showed that relapse-free survival (RFS) was linked to *CCND1* status (*P*=0.011; Figure 1

). The RFS of the 15 patients with *CCND1*-amplified tumours (5-year RFS 63.0% (36.6–89.4); RR=0.37 (0.15–0.87)) and of the 32 patients with *CCND1*-unamplified-overexpressed tumours (5-year RFS 93.8% (85.4–100); RR=1.8 (0.7–4.0)) were respectively shorter and longer than the RFS of the 87 patients without *CCND1* alterations (5-year RFS 80.7% (72.2–89.2); RR=1). The prognostic significance of *CCND1* mRNA and DNA status persisted for lymph-node-negative (*P*=0.022) but not for lymph-node-positive patients (*P*=0.13). Using a Cox proportional hazards model, we also assessed the prognostic value, for RFS, of parameters that were significant in univariate analysis, i.e. lymph-node status (Table 1) and *CCND1*-unamplified-overexpression and *CCND1*-amplification status (Figure 1). The prognostic significance of lymph-node and *CCND1*-unamplified-overexpression status only persisted in Cox multivariate regression analysis ( Table 5

**Table 5:**
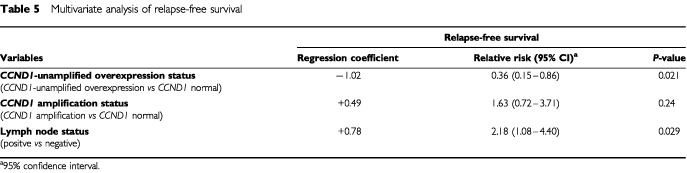
Multivariate analysis of relapse-free survival

). The adjusted relative risk associated with these two parameters, taking into account menopausal status, macroscopic tumour size, histological grade and steroid receptor status, did not change their prognostic significance for RFS (data not shown).

### Relationship between *CCND1* mRNA and DNA status and *MYC, ERBB2* and *RB1* expression status

Because alterations in any component in the cell cycle regulatory *p16/CCND1/RB1* pathway may have similar oncogenic effects, we studied the relationship between abnormalities of the *CCND1* gene and altered expression of the *RB1* gene, which had already been tested at the mRNA level (Bièche and Lidereau, 2000). We observed no correlation (or a negative correlation) between *CCND1* mRNA and/or DNA alterations and *RB1* underexpression (Table 4).

We also observed no link between *CCND1* gene abnormalities and altered mRNA expression of the *MYC* and *ERBB2* genes (Table 4).

## DISCUSSION

The aim of this study was to assess the prognostic significance of *CCND1* status at both the RNA level (to identify overexpression) and the DNA level (to identify gene amplification) in 134 unilateral invasive primary breast tumours with a known long-term outcome. The frequencies of *CCND1* amplification (11.2%) and overexpression (33.6%) are in agreement with those previously reported in the literature (Ali *et al*, 1989; Borg *et al*, 1991; Schuuring *et al*, 1992; Henry *et al*, 1993; McIntosh *et al*, 1995; Gillett *et al*, 1996; Barbareschi *et al*, 1997; Jares *et al*, 1997; Nielsen *et al*, 1997; Kenny *et al*, 1999). Joint analysis of the *CCND1* gene at both the mRNA and DNA levels showed that patients with a good outcome had *CCND1*-unamplified-overexpressed tumours while those with a poor outcome had *CCND1*-amplified tumours. Our results confirm the poor outcome associated with *CCND1* amplification (Ali *et al*, 1989; Borg *et al*, 1991; Schuuring *et al*, 1992; Henry *et al*, 1993). More interestingly, they suggest that *CCND1* amplification status should be taken into account when studying the prognostic significance of *CCND1* overexpression. Indeed, the good outcome of patients with *CCND1*-overexpressing tumours was reverted by *CCND1* amplification. This may explain why some authors have linked *CCND1* overexpression to good outcome (Gillett *et al*, 1996; Nielsen *et al*, 1997), while others to poor outcome (McIntosh *et al*, 1995; Kenny *et al*, 1999).

These data on the *CCND1* gene status obtained at both the RNA and DNA levels shed light on several important questions.

### The mechanisms underlying *CCND1* overexpression in unamplified tumours

These data confirm our previous report from a small series of breast tumours (Spyratos *et al*, 2000) where *CCND1* overexpression is strongly linked to oestrogen receptor positivity. However, is *CCND1* overexpression a cause or a consequence of transcriptional activation of oestrogen receptors in breast tumour cells? Previously reported *in vitro* data suggest that *CCND1* overexpression is dependent on the presence of oestrogen and oestrogen receptors, and that anti-oestrogens inhibit cyclin D1 expression in breast cancer cells (Sutherland *et al*, 1997). However, no oestrogen response element (ERE) has been identified in the *CCND1* promoter. Sabbah *et al* (1999) recently suggested a mechanism by which ER regulates *CCND1* gene transcription through a cyclic AMP response element (CRE). Alternatively, there is increasing evidence that cyclin D1 forms a direct complex with the oestrogen receptor and can regulate this transcriptional activity without the need for oestrogen (Neuman *et al*, 1997; Zwijsen *et al*, 1997; McMahon *et al*, 1999). Zwijsen *et al* (1997) observed direct physical binding of cyclin D1 to the hormone-binding domain of the oestrogen receptor, resulting in increased binding of the receptor to oestrogen response-element sequences and upregulating oestrogen receptor-mediated transcription. Activation of the oestrogen receptor by cyclin D1 is independent of complex formation to a CDK partner but necessitates the recruitment of p300/CREB-binding protein-associated protein (P/CAF) and steroid receptor coactivator-1 (SRC-1), and is not inhibited by anti-oestrogens. Wilcken *et al* (1997) showed that tamoxifen inhibition of cell progression was overcome in both T-47D and MCF7 cells when cyclin D1 expression was ectopically induced. The influence of cyclin D1 status on the response to oestrogen and anti-oestrogens such as the tamoxifen warrants further study.

### The good outcome of patients with *CCND1* overexpressing tumours

One simple explanation is that *CCND1* overexpression is associated with well-differentiated, oestrogen receptor-positive tumours (which are known to have a more favourable prognosis and a respond better to anti-oestrogen therapy. Alternatively, it may be due to more rapid cell proliferation and, thus, greater chemosensitivity. The possible relation between *CCND1* overexpression and the response to chemotherapy could not be studied in this retrospective series of unselected patients because the treatments used after surgery were highly variable. To test this hypothesis, it will be necessary to conduct a prospective randomized clinical study to show that CCND1 overexpression do influence outcome only in patients who received chemotherapy as compared to untreated patients.

### The possible involvement of the *CCND1* gene in 11q13 amplicon selection and the poorer outcome of patients with 11q13-amplified tumours

The more plausible explanation is that *CCND1* has a true role of oncogene and it is a more important gene as a driving force for 11q13 amplification. Amplification at 11q13 leads to higher *CCND1* expression, resulting in more rapid proliferation of epithelial breast tumours and, thus, in poorer outcome.

However, three of the 15 11q13-amplified tumours were examined had not *CCND1* overexpression (Table 3). Similar breast tumours have been observed by several other authors (Gillett *et al*, 1994; Barbareschi *et al*, 1997). Moreover, we previously showed that *CCND1* mRNA overexpression is not related to a proliferative marker, the S-phase fraction, measured by flow cytometry (Spyratos *et al*, 2000).

Conversely, the amplification unit on chromosome 11q13 may encompass other gene(s) that could be the major 11q13 amplicon selector and whose overexpression might contribute to poor clinical outcome. One candidate is the oncogene *EMS1*, which is located approximately 800 kb telomeric to *CCND1* and encodes an cytoskeletal actin-binding protein. It is amplified and overexpressed independently of *CCND1* and oestrogen receptor expression, and, in contrast to cyclin D1, is not regulated by oestrogen (Hui *et al*, 1998). Thus, *CCND1* overexpression could be due exclusively to the presence of oestrogen receptors, and the higher *CCND1* overexpression observed in amplified tumours than in overexpressed-unamplified tumours could be due to a simple gene dosage effect. In agreement with this hypothesis, the three 11q13-amplified tumours with *CCND1* normal expression were oestrogen receptor-negative (Table 3). However, it should be noted that we also observed one oestrogen receptor-negative tumour with *CCND1* overexpression and amplification (Tumour CCND1209; Table 3).

Finally, 11q13 amplification may simply reflect genomic instability in breast tumours. Coquelle *et al* (1998) showed a key role of hypoxia in inducing breaks at fragile sites and initiating intrachromosomal amplification. Such fragile sites (FRA11A and FRA11F) are located on each side (centromeric and telomeric) of the 11q13 amplicon.

In conclusion, we observed a major link between *CCND1* mRNA status and ER status, confirming a role for the *CCND1* gene as an ER-responsive gene or ER-coactivator gene in breast cancer. *CCND1* amplification might simply be an additional mechanism contributing to high levels of *CCND1* overexpression observed in oestrogen receptor-positive tumours, through a simple gene dosage effect. These findings, together with the observation of several oestrogen receptor-negative tumours with 11q13 amplification but no CCDN1 overexpression, challenge *CCND1* gene as the main 11q13 amplicon selector.

*CCND1* may serve as a molecular marker for predicting hormone responsiveness in breast cancer, as hypothesis currently being tested in a large and homogeneous clinical study.
